# Evolution of metabolic network organization

**DOI:** 10.1186/1752-0509-4-59

**Published:** 2010-05-11

**Authors:** Aurélien Mazurie, Danail Bonchev, Benno Schwikowski, Gregory A Buck

**Affiliations:** 1 Institut Pasteur, Systems Biology Lab, Department of Genomes and Genetics, F-75015 Paris, France; 2 CNRS URA 2171, F-75015 Paris, France; 3 Center for the Study of Biological Complexity, Virginia Commonwealth University, Richmond VA 23284, USA; 4 Department of Mathematics and Applied Mathematics, Virginia Commonwealth University, Richmond VA 23284, USA; 5 Department of Microbiology and Immunology, Virginia Commonwealth University, Richmond VA 23298, USA

## Abstract

**Background:**

Comparison of metabolic networks across species is a key to understanding how evolutionary pressures shape these networks. By selecting taxa representative of different lineages or lifestyles and using a comprehensive set of descriptors of the structure and complexity of their metabolic networks, one can highlight both qualitative and quantitative differences in the metabolic organization of species subject to distinct evolutionary paths or environmental constraints.

**Results:**

We used a novel representation of metabolic networks, termed network of interacting pathways or NIP, to focus on the modular, high-level organization of the metabolic capabilities of the cell. Using machine learning techniques we identified the most relevant aspects of cellular organization that change under evolutionary pressures. We considered the transitions from prokarya to eukarya (with a focus on the transitions among the archaea, bacteria and eukarya), from unicellular to multicellular eukarya, from free living to host-associated bacteria, from anaerobic to aerobic, as well as the acquisition of cell motility or growth in an environment of various levels of salinity or temperature. Intuitively, we expect organisms with more complex lifestyles to have more complex and robust metabolic networks. Here we demonstrate for the first time that such organisms are not only characterized by larger, denser networks of metabolic pathways but also have more efficiently organized cross communications, as revealed by subtle changes in network topology. These changes are unevenly distributed among metabolic pathways, with specific categories of pathways being promoted to more central locations as an answer to environmental constraints.

**Conclusions:**

Combining methods from graph theory and machine learning, we have shown here that evolutionary pressures not only affects gene and protein sequences, but also specific details of the complex wiring of functional modules in the cell. This approach allows the identification and quantification of those changes, and provides an overview of the evolution of intracellular systems.

## Background

Networks are commonly used in biology to represent molecular mechanisms occurring in the cell, from protein-protein interactions to enzymatic reactions (metabolic networks) and gene regulations (gene regulatory networks). The use of tools derived from graph theory has led to the successful characterization of some aspects of the topology and organization of these networks and the intracellular mechanisms they represent. The study of intracellular networks so far has revealed their small-worldness [1] and scale-freeness [2], as well as the existence of network motifs, [3,4] and a strongly modular organization [5-8]-see Barabasi *et al*. [9] for a review. Network representations are also increasingly used to characterize the function of the objects (genes, proteins, metabolites) they interconnect. This has lead to applications such as inference of functional annotation [10] or predictions of new disease-related or longevity-related genes [11,12]-see Aittokallio *et al*. [13] for a review.

Networks are also used in comparative studies to highlight the differences and similarities existing in the organization of the intracellular mechanisms of multiple species. Studies have been published that compare the topological features of metabolic networks for taxa sampled from all three kingdoms of life [14-17]. These investigations shed light on how these metabolic networks differ among the archaea, bacteria and eukarya, as a result of natural selection processes [18]. The results show that while some properties are shared by all taxa (e.g., their metabolic networks are all scale-free), bacteria species distinguish themselves over archaea and eukarya by having a shorter network average path length [15,16]. Metabolic networks of archaea species also exhibit a lower average clustering coefficient, betweenness centrality and scale-freeness [17] than those of other species. Finally, the organization of the metabolism of species appears to be impacted by the species lifestyle and phenotype; e.g., by the variability of their habitat [19], or their optimal growth temperature [20]. Metabolic networks of modern organisms have also been compared to inferred ancestral ones [21]. These studies indicate that niche specialization and extreme environments tend to decrease the modularity of the metabolic networks of taxa.

A comparison of intracellular networks on the basis of their topological similarity is thus a promising approach to study the evolution of species. It complements comparative genomics strategies that focus on comparison of gene sequence and genome architecture. However, while metabolic networks are a useful representation of the metabolic capabilities of the cell, they are of low organizational level, i.e., at the level of individual enzymatic reactions. We propose here to proceed from higher-level organizational principles of cell metabolism, and determine how these principles change under evolutionary pressures.

## Results and Discussion

### Comparison of metabolism organization

Proceeding from publicly available annotations, different groups of taxa were selected for this study as representative of environments or lineages of particular evolutionary interest. Hence, the organization of the metabolism of taxa from the three kingdoms archaea, bacteria and eukarya was compared, with an additional comparison of the superkingdom prokarya and the kingdom eukarya. Unicellular eukarya were opposed to multicellular eukarya to reveal how the more stable environment and cellular differentiation that a multicellular body implies impacted the organization of metabolism. Free-living bacteria were compared to host-associated bacteria, as taxa from the latter group typically inhabit less demanding environments-the host providing most nutrients, while limiting competitors and predators. Immotile bacteria were compared to motile ones, motility being a key competitive advantage allowing an active search for nutrients and evasion from predators or harmful environments. Aerobic bacteria were compared to anaerobes and to facultative aerobic bacteria as a potential negative control; in Raymond *et al*. [22], the authors demonstrated that aerobic metabolism had little impact on the rewiring of metabolic network structure during evolution, although network size was affected. Finally, bacteria living in environments of distinct salinity (halophiles versus halotolerants) and temperature (mesophiles versus psychrophiles and thermophiles) were compared, to assess any large-scale adaptation of their metabolic network to these environments. Each group of taxa was thus selected for being a result of evolutionary pressures, and opposed to other groups either lacking their most prominent characteristics, or subject to distinct evolutionary pressures.

Networks of interacting pathways (NIPs) were constructed to represent the high-level organization of cellular metabolism in all taxa as described in Mazurie *et al*. [23], using the latest version of the KEGG metabolic reactions database [24]. Then, for all taxa group comparisons considered, classification models were built to assess the effectiveness of a comprehensive set of 52 quantitative descriptors of the structure and complexity of NIPs in distinguishing the groups. Briefly, classification models were trained to predict the group to which a taxon belongs solely from the values of its NIP descriptors. Good predictions, as assessed by the accuracy and Kappa statistics of 10-fold cross-validation, would indicate that a sufficiently large difference exists between the NIPs of the different taxa groups to allow an accurate classification. Results are shown on Table 1.

**Table 1:** Classification models performance.

Comparison	Accuracy	Kappa statistic	Classification model
Archaea (56) vs. Bacteria (600) vs. Eukarya (87)	93.54% (89.50%)	0.81 (0.62)	Functions.Logistic
Prokarya (656) vs. Eukarya (87)	98.25% (96.90%)	0.91 (0.84)	Functions.MultilayerPerceptron
Unicellular (44) vs. Multicellular (43) Eukarya	96.55% (96.55%)	0.93 (0.93)	Rules.JRip
Free-living (525) vs. Host-associated (61) Bacteria	91.98% (92.32%)	0.45 (0.48)	Rules.OneR
Immotile (202) vs. Motile (322) Bacteria	72.33% (72.14%)	0.40 (0.40)	Lazy.IB1
Anaerobe (253) vs. Facultative aerobe (170) vs. Aerobe (253)	61.20% (57.92%)	0.38 (0.33)	Trees.RandomForest
Halotolerant (4) vs. Halophile (15) Bacteria	78.95%	0.00	Functions.LibSVM
Psychrophile (24) vs. Mesophile (508) vs. Thermophile (61) Bacteria	86.00%	0.04	Functions.LibSVM

**Figure 1:**
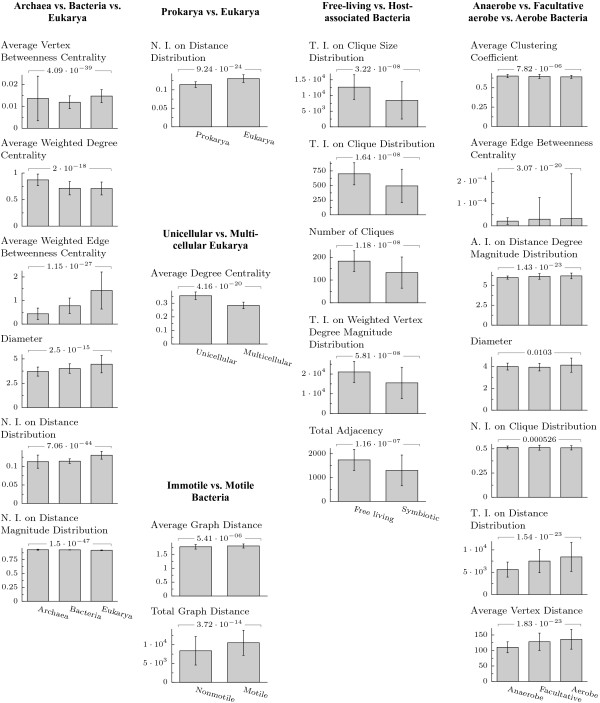
**Values of the NIP descriptors (abridged)**. For each group comparison, the descriptors of the structure and complexity of NIPs reported are those shown to best discriminate the different groups of taxa considered. Bars represent the average value and standard deviation of a given descriptor for each group of taxa, based on metabolic networks extracted from KEGG. The hypothesis that the descriptor value is the same over all groups was evaluated for each metabolic dataset either by a Kruskal-Wallis test (comparisons of three groups) or Mann-Whitney U test (comparisons of two groups). Resulting p-values were corrected for multiple testing using Bonferroni correction. Values for all 52 descriptors are available in Additional file 1. A.I., N.I., T.I.: Average, Normalized and Total Information, respectively.

Scores obtained by training classification models to discriminate groups of taxa based on quantitative descriptors of the structure and complexity of their Networks of Interacting Pathways (NIPs). Data given is the number of taxa considered for each group, as well as the accuracy and Kappa statistics on 10-fold cross-validation of the best performing classification model when using all 52 NIP descriptors and, in parentheses, those obtained with the best subsets of descriptors identified (see Figure 1).

The high scores obtained for most of the taxa group comparisons confirm that distinct evolutionary pressures are reflected by distinct organizational principles of the metabolism of species. Our results confirm the finding from Raymond *et al*. [22] showing a lack of significant differences between metabolic networks of aerobic versus anaerobic species. We also show that no distinction could be made between bacteria living in an environment of distinct salinity or distinct temperature, based on the topology of their NIPs. In the first case, this could be explained by a lack of representative taxa for halotolerant bacteria (only 4, compared to 15 halophiles). In the second case, the large number of representative taxa suggests a genuine absence of significant differences in the structure and complexity of NIPs of psycrophilic, mesophilic and thermophilic bacteria.

### Significant changes of metabolism organization

We first investigated the differences in the structure and complexity of the NIPs of species from different lineages or environments. Among the 52 NIP descriptors used for this investigation, we identified using feature selection algorithms (see Methods) the smallest subsets of descriptors that best discriminate taxa. The scores obtained are reported in Table 1. A list of these best discriminating descriptors of metabolic organization, as well as the average value obtained for each group of taxa, is given in Figure 1; values for all descriptors are provided as Additional file 1.

#### Prokarya versus eukarya

The transition from archaea and bacteria to eukarya is associated with an increase in the NIP size as revealed by the network radius, diameter, and the average distance between pathways. The weighted edge and vertex betweenness centrality (fraction of all metabolic exchanges going through a pathway) also increase, revealing not just larger but also more efficiently organized NIPs. Thus, eukarya species have a hierarchically organized metabolism, with more central pathways located at the cross-points of many pathway pair communications. In contrast, the average pathway connectivity (number of pathways a pathway is connected to) decreases from prokarya to eukarya: bacteria species have denser NIPs than both archaea and eukarya species as shown by the total adjacency, average node degree, and connectedness (or density). The higher density of bacterial NIPs also results in higher local clustering-larger number and size of cliques (subsets of pathways that are completely inter-connected)-and higher weighted clustering coefficient, showing more intensive pathway cross-talk in bacteria than in eukarya species. It should be noted that due to the fact that archaea annotations are the most recent and likely incomplete, our conclusions concerning this kingdom should be considered preliminary.

The denser, but less organized metabolism of prokarya is likely a consequence of higher evolutionary pressures in these species, whose metabolic organization needs to adapt to a greater variety of environments than that of eukarya. Indeed, prokarya have a higher rate of horizontal gene transfer than eukarya [25]. Higher genome plasticity means less time to integrate newly acquired metabolic capabilities with existing ones [26,27]. However, high plasticity allows fast responses to challenges from the environment, when possessing a given metabolic capability is more important for the survival than its fine integration in the cell.

We compared some of our results with those of Ma *et al*. [15,16] and Zhu *et al*. [17]. A direct comparison is not possible, as the cited authors investigated the entire network of biochemical reactions-which is sparse-instead of the more abstract and denser NIP, which reflects the high-level organization of these reactions. Also, our study is based on more recent metabolic datasets, with a larger set of taxa. Yet, a comparison of the patterns in both types of networks has its own merit. Our results do not confirm the finding from Ma *et al*. about the metabolic network of eukarya and archaea species having a longer average path length (or average network distance) and lower OCCI (overall closeness centralization index [28]) than those of bacteria. Rather, we show a pattern of increasing average network distance from archaea to bacteria to eukarya, with values of 1.7, 1.8 and 1.9, respectively for their NIPs. We observe a similar increase of the NIP diameter with values of 3.7, 4.0 and 4.5. The average closeness centrality, which is calculated from reciprocal distances, follows the opposite trend with values of 0.61, 0.58 and 0.55. A comparison with the OCCI descriptor used in Ma *et al*. [16] is not possible, however, due to the very different definition of the two indices [28]. We also do not confirm for NIPs the result of Zhu *et al*. [17] showing the metabolic networks of twelve archaea species as exhibiting lower average clustering coefficient and betweenness centrality than those of bacteria and eukarya species. Our result for NIPs averaged over 56 archaea species shows they have the highest clustering coefficient (with values of 0.68, compared to 0.64 and 0.63 for bacteria and eukarya, respectively), and intermediate betweenness centrality values (0.014, compared to 0.012 and 0.015). One may suppose that besides the much higher connectivity of NIPs, the considerably larger set of archaea in our study perhaps contributes to these essentially different patterns.

#### Unicellular versus multicellular eukarya

The transition from unicellular to multicellular eukarya is associated with a larger NIP (as shown by the increased number of vertices and edges, increased diameter, average network distance, and average node distance, respectively) with more central pathways (as shown by the increased weighted betweenness centrality). However, two other aspects of pathway centrality are found to be less important in multicellular eukarya: the degree centrality (fraction of the network a pathway is connected to), and the closeness centrality (inverse of the distance between a pathway and all other pathways). The decrease in degree centrality is independent of the network size, and is a consequence of the smaller network connectedness. The decrease of closeness centrality is a consequence of the increased distance between pathways in the network. Thus, we demonstrate that during the transition from unicellular to multicellular eukarya evolution preferentially selected pathways with high betweenness centrality, which acts as switchboards for the intracellular metabolites trafficking. Less favored were pathways directly connected with many other pathways, and pathways with a short distance to other pathways.

Finally, we observe similar values for the average pathway connectivity and local clustering in unicellular and multicellular eukarya as shown by the weighted and unweighted average node degrees, and the average weighted clustering coefficient and average information on the distribution of the number and size of cliques, respectively. With unicellular eukarya preceding multicellular eukarya, it appears that these two aspects of the metabolic network topology have been conserved during evolution as optimal organizational principles.

#### Free living versus host-associated bacteria

As expected, the transition from free living to host-associated bacteria is reflected by a smaller NIP. There are fewer pathways, fewer connections between pathways and fewer metabolites exchanged, as shown by the lower values of the weighted NIP descriptors-which not only takes into account the existence of links between pathways, but also the number of metabolites that are exchanged. We demonstrate here that those descriptors are crucial in comparing NIPs. Accounting in a more complete manner for the cross-talk between pathways, weighted descriptors increase the reliability of the identified pattern of change. This is best illustrated by weighted betweenness centrality and weighted clustering coefficient, which reverse the trend of change shown by their unweighted analogues from increasing to decreasing. The most sensitive descriptors discriminating free living from host-associated bacteria are those related with local clustering as reflected in the number, size and distribution of cliques (Figure 1). Another group of descriptors with high discriminative power are those related to connectivity (Additional file 1). Thus, the metabolism of host-associated bacteria is sparser, and contains less 'hubs' (pathways with high connectivity), as shown by the smaller range of vertex degree values.

Our results are consistent with the facts about bacteria living under stable environments, such as within a host body. They undergo major genome reduction [29], and the host tissues provide a constant supply of metabolites, eliminating the need to maintain the corresponding pathways. We show here that the adaptation to a parasitic or symbiotic lifestyle also affects the degree of organization of cellular metabolism, resulting in a less complex network and less communication between metabolic pathways. A similar process has been observed for gene regulatory networks, with transcription factors-the key organizers of the genes transcriptional activity-being preferentially lost during the adaptation to a host-associated lifestyle [30]. In both cases the evolution toward a less organized network certainly reflects the adaptation to a less demanding and more static environment. Indeed, varying environments have been shown, at least *in silico*, to favor the re-use of existing metabolisms [19], which can only be achieved through a denser and more centrally organized wiring of metabolic pathways.

#### Immotile versus motile bacteria

The transition from an immobile lifestyle to a mobile one is a major change, which causes metabolism reorganization similar to the evolutionary leap from unicellular to multicellular species. Thus, motile bacteria have more metabolic pathways, i.e., they are characterized by NIPs having more vertices and edges, more (and larger) cliques, larger average distance between pathways, and larger ranges of vertex degrees and vertex distances than immotile bacteria. Their metabolic network is also more organized, with more central pathways as shown by the weighted edge and vertex betweenness centrality descriptors. Yet, motility does not increase the diameter of the network or the average closeness centrality, and it does not change the network clustering.

**Figure 2:**
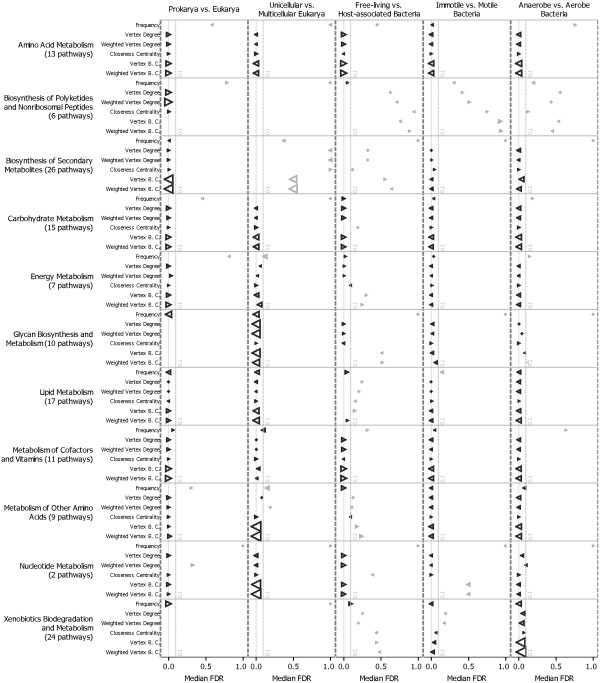
**Effects of lineage and environment on pathway frequency, connectivity and centrality**. The amplitude of variation of six scores of frequency, connectivity and centrality, and a p-value evaluating the significance of this variation are reported for all metabolic pathways. P-values were calculated by either a Fisher's Exact Test (frequency) or Mann-Whitney U-text (connectivity and centrality), and corrected for multiple testing using the Benjamini-Hochberg method [31]. The median variation and the p-value for pathways in the same functional category were pictured as either a triangle or a diamond. A triangle pointing left (◀) means the score increases from left to right (e.g., from Prokarya to Eukarya), while a triangle pointing right (▶) means the score decreases from left to right. A diamond means the score does not change. The position of the symbol is proportional to the median corrected p-value, i.e. False Discovery Rate, and its size is proportional to the amplitude of variation. All values are available in Additional file 2. B.C.: Betweenness Centrality.

Motile bacteria can forage for food (sugars and amino acids), and are generally attracted or repelled by various stimuli in their environments (e.g., chemotaxis). We show here that the metabolism of motile bacteria evolved to be bigger and more organized than those of immotile bacteria. As shown in Figure 2, the pathways that most benefits from this higher organization are those related to amino acid and carbohydrate metabolism. Although the characterization of the exact link between chemotaxis and a centrally organized metabolism would deserve additional investigations, this result is consistent with the increased ability for motile bacteria to sense a shortage of intracellular nutrients, and to process extracellular ones.

#### Anaerobic versus aerobic bacteria

We compared the metabolic organization of bacteria living in anaerobic, facultative aerobic and aerobic conditions. Overall, aerobes and facultative aerobes have larger metabolic networks than anaerobes, as shown by the number of vertices, edges, and total graph distance. The characteristics of the facultative aerobes are found to be closer to those of the aerobes than the anaerobes. However, few differences exist among the three groups in terms of the vertex degree, distance, degree centrality or closeness centrality descriptors. Thus, while aerobic conditions enlarge the metabolic network, its organization remains basically the same, confirming the results from Raymond *et al*. [22]. The only partial reorganization is an increase of the average weighted betweenness centrality, showing that some pathways acquired a more central position in the network.

### Significant changes in pathway importance

We next asked the question of how the importance of pathways changed during the evolution of species. More specifically, we are interested in pathways that are acquired or lost, those evolving to be more (or less) connected to other pathways in the cell, and finally those evolving to be more (or less) centrally located in the NIPs.

Thus, we calculated a frequency score for each metabolic pathway together with five descriptors of pathway connectivity and pathway centrality. Frequency is the fraction of taxa in a given subset that possess this pathway. Connectivity is represented by the weighted and unweighted versions of vertex degree; i.e., the number of connections with other pathways, and the number of metabolites exchanged. Centrality is represented by the closeness centrality and the weighted and unweighted versions of betweenness centrality; i.e., distances to other pathways, and fraction of the pathways cross-talk passing through. Closeness centrality may be regarded as a measure of how long it will take in average for metabolites to spread (through successive enzymatic conversions) from a pathway to other reachable pathways in the NIP: the higher the value, the shorter time needed. Betweenness centrality is a measure of how much of the metabolite traffic in the cell goes through a given pathway. High values reveal more efficiently organized metabolic networks, with central pathways being reused in, and in control of, a multitude of metabolic processes.

We tested the null hypothesis that the values taken by these scores are not significantly different across subsets of taxa. For each score and pair of subsets a p-value was calculated (using the Fisher's exact test for the frequency score, and Mann-Whitney U-test for the scores of connectivity and centrality) together with the amplitude of variation of the score. Those p-values were corrected for multiple testing using the Benjamini-Hochberg method [31]. Finally, the median variation and p-value for pathways in the same functional category (as defined by KEGG) was extracted. The results are shown in Figure 2 and in Additional file 2. From those we made the following observations:

- Pathways related to lipid metabolism are better represented and integrated in the metabolism of multicellular eukarya than unicellular eukarya. In the case of vertebrates, this evolution certainly allowed the apparition of complex signal transduction systems based on products of lipid metabolism, like steroid hormones [32].

- Pathways related to glycan metabolism are more frequent and more integrated in the metabolism of multicellular eukarya than unicellular eukarya. This can be explained by the importance of extra-cellular matrix, of which proteoglycans are an important structural component, for the formation of multicellular structures [33]. It also supports the importance of glycosylation as a key mechanism for cell-cell communications and interactions, as shown by the fact that nearly all membrane and extracellular proteins are glycosylated [34]. Finally, this result is consistent with some of the findings from Peregrin-Alvarez *et al*. [35] showing a higher conservation of glycan-related pathways in eukarya.

- Similarly, pathways related to glycan metabolism are more integrated in the metabolism of motile compared to immotile bacteria. This supports the importance of glycans as a motility factor [36,37]. Motile bacteria also have a higher integration of pathways related to the biodegradation of xenobiotics than immotile bacteria. Xenobiotics are poisons in the surrounding or inside the bacteria cell. Such metabolic capability is certainly advantageous for the motile cell, allowing it to actively colonize polluted environments with few or no competitors.

- The increase of metabolic capabilities associated to the transition from an anaerobic to aerobic lifestyle [22] essentially benefited to pathways related to lipid, amino acids and xenobiotics degradation metabolism. These pathways are also more connected and more integrated, especially so for xenobiotic degradation. This better integration certainly results from the cross-talks between many pathways that oxidative processes provide. E.g., the oxidation of amino acids and lipids to produce energy or intermediary metabolites, or the oxidation of xenobiotics [38] as exemplified by the cytochrome oxidase family.

- The decrease of NIP density observed when transitioning from free living to host-associated bacteria appears to be due in part to a significant decrease in the frequency of pathways related to carbohydrate and energy metabolism. Also, there is a general decrease of connectivity and centrality of all pathways. This is consistent with results from Henrissat *et al*. [39], showing that the lack of glycogen metabolism is a trait associated with parasitic behavior in bacteria.

Finally, we report two observations, which we leave open for possible interpretation. We observed a divergence between change of centrality and connectivity for pathways related to amino acid and carbohydrate metabolism when comparing unicellular and multicellular eukarya, and pathways related to the biosynthesis of secondary metabolites when comparing prokarya and eukarya. These pathways are less directly connected to the rest of the metabolic network in the latter groups, while being more central. It means that these pathways process metabolites from and to a smaller range of neighboring pathways, while tunneling more of the overall metabolic fluxes in the cell.

## Conclusions

Addressing the effect of evolutionary pressures on the metabolism of species, we used a higher representation of metabolisms-the Network of Interacting Pathways, or NIP-focusing on the logic of metabolic organization, rather than on the details of the underlying molecular mechanisms. In this study we demonstrate that specific aspects of the structure and complexity of these NIPs are discriminative of species from different lineages and lifestyle; i.e., species under distinct evolutionary pressures.

We found that species that evolved to accommodate more complex lifestyles; e.g., eukarya compared to prokarya, multicellular eukarya compared to unicellular eukarya, motile bacteria compared to immotile bacteria, developed not only bigger but also more structured metabolic networks. Communication between pathways is typically enhanced by the addition of hubs (highly connected pathways, which convert metabolites for a broader range of input and output pathways) and switchboards (centrally connected pathways, which capture a large fraction of the whole metabolite traffic). As for the transition from anaerobic to aerobic bacteria, we observed only enlargement of networks with the inclusion of additional metabolic pathways, while the organization and complexity of the cross talk between pathways remained mostly unchanged. The reorganization of metabolic networks is shown to be heterogeneous, with specific categories of pathways being promoted to more central or connected locations. Thus, we demonstrated the increased importance of lipid metabolism for multicellular eukarya, glycan and xenobiotics metabolism for motile bacteria, and lipid, amino acid and xenobiotics metabolism for aerobic bacteria. Conversely, we found that the decrease in complexity of the metabolism of host-associated bacteria significantly impacted carbohydrate and energy metabolism.

The unique combination of methods from graph theory, information theory, and machine learning used for this study enabled the identification of statistically significant differences between the NIPs of species, as well as the quantification of the relation between these differences and the species lineage or lifestyle. The same method could as well be applied to other types of network-based representations of species, as long as enough experimental data are available to represent groups of species of interest. The rapidly growing collection of protein interaction networks being published suggests this dataset could be the next logical subject of study to track how evolution shaped the molecular biology of cells.

## Methods

### Network of interacting pathways

Metabolic reactions for 1143 species were retrieved from January 2010 release of the KEGG database [24] using the KEGG API. All currency metabolites (except water) were kept, based on the conclusion from Mazurie *et al*. [23] that those common metabolites improve the capability of metabolic networks to capture the phylogeny of species. Out of these 1143 species, 1042 were selected as having completely sequenced genomes according to GOLD 3.0 [40], and a consistent metabolic pathway annotation. Consistency of pathway annotation was evaluated by comparing the number of pathways for each species to the logarithm of its number of ORFs. The resulting correlation was significant (r^2 ^of 0.66, p-value below 2.2 × 10^-16^). All species with a number of pathways below average (species in the lowest 5% residuals) were filtered out. Finally, these 1042 species were clustered into 743 taxa to account for strains of the same species, subspecies, pathovars and biovars. For each of these 743 taxa, Networks of Interacting Pathways (NIPs) were reconstructed by linking overlapping metabolic pathways sharing at least one metabolite. I.e., two pathways are connected if the enzymes in the first pathway consume or produce at least one metabolite that is produced or consumed in the second pathway. The resulting links between pathways were weighted by the number of metabolites exchanged [23].

### Groups of taxa

Based on the annotations from GOLD 3.0 [40] the 743 taxa were separated into the following groups: 656 prokarya (56 archaea and 600 bacteria) and 87 eukarya, 44 unicellular and 43 multicellular eukarya, 253 aerobe, 126 anaerobe and 170 facultative aerobe bacteria, 322 motile and 202 immotile bacteria, 4 halotolerant and 15 halophile bacteria, 525 free-living and 61 host-associated bacteria, and 24 psychrophile, 508 mesophile, and 61 thermophile Bacteria. All annotations are available in the Additional file 3.

### Network descriptors

Four categories of quantitative descriptors of network structure and complexity, based on the notions of degree, centrality, distance and cliques, were used to characterize the NIPs constructed to represent taxa (see Additional file 4 and associated references for details on the 52 descriptors used-25 basic and 27 derivatives). This set includes descriptors that have been successfully used in other fields [41-46], as well as others specifically developed for the needs of our study. The latter include several weighted descriptors characterizing the intensity of cross talk between the metabolic pathways; they present a more realistic picture of network connectivity, distances, paths, and centrality at the higher organizational level of NIPs. Complete subgraphs (cliques) and their distribution were another addition to the basic topological characteristics of metabolic networks. The extensive set of network descriptors devised for this study thus enables the covering of all potentially relevant features of intracellular networks when comparing taxa. The values of these descriptors were calculated for each NIP using the NetworkX library https://networkx.lanl.gov/.

### Machine learning

For each taxa group comparison a training set was constructed by reporting for each taxa the values taken by all 52 descriptors of its NIP together with the group it belong to. These training sets are available as Additional file 5. Supervised learning algorithms implemented in the Weka toolbox (Witten *et al*. [47] and Additional file 6) were applied on the training sets to predict the taxa group membership from the NIP descriptors values. A score of accuracy and Kappa statistic of the 10-fold cross-validation and that of the whole training set were calculated by comparing known and predicted membership. For a given training set, the accuracy and Kappa statistic were then taken as the highest obtained among all classification models. A high accuracy and Kappa statistic would mean the taxa group membership could be predicted from the NIP's high-level organization of metabolic networks. The smallest subset of NIP descriptors that still performs as well as the complete set of 52 was identified using feature selection algorithms [48,49] and a heuristic evaluation of subsets of descriptors on the classification models identified earlier as the best ones. A tool, MetaClassify, was developed to automate the training of the classification models and to retrieve the results http://oenone.net/tools/.

## Authors' contributions

AM carried out the NIPs reconstruction, topological descriptors calculation, machine learning experiments and drafted the manuscript. DB designed the topological descriptors. AM and DB devised the study and interpreted the results. All authors read, discussed, and approved the final manuscript.

## Supplementary Material

Additional file 1 **Values of the NIP descriptors**. The average value and standard deviation of all 52 NIP descriptors is calculated for all taxa of each group considered in the study. Those NIP descriptors revealed as the best discriminative for a given taxa groups comparison are highlighted in bold. P-values are obtained from Kruskal-Wallis one-way analysis of variance (comparisons of three groups) or Mann-Whitney U-test (comparisons of two groups), and corrected for multiple testing using Bonferroni correction. Click here for file

Additional file 2 **Effects of lineage and environment on pathways frequency, connectivity and centrality**. The variation of six scores of frequency, connectivity and centrality, and the p-value evaluating the significance of this variation (calculated by either a Fisher's exact or Mann-Whitney U-test) are reported for each metabolic pathway. P-values are corrected for multiple testing using the Benjamini-Hochberg method. Click here for file

Additional file 3 **Groups of taxa. **List of the 743 taxa considered in this study, sorted per group of common lineage, environment or life-style, together with the corresponding KEGG identifiers. Click here for file

Additional file 4 **Network descriptors. **Topological and information-theoretic network descriptors used in this study. Click here for file

Additional file 5 **Training sets. **NIP descriptor values and group membership of the 743 taxa considered in this study. Click here for file

Additional file 6 **Classification models. **List of the classification models with the best performance in discriminating taxa groups based on NIP descriptor values. Click here for file
