# Supplementary material for: Evolution of metabolic network organization
Source: BMC Syst Biol. 2010 May 11;4:59. doi: 10.1186/1752-0509-4-59 (PMC2876064; doi:10.1186/1752-0509-4-59)
Supplement: Additional file 4 — Network descriptors. Topological and information-theoretic network descriptors used in this study. [file 1752-0509-4-59-S4.PDF]

Supplementary file: Network descriptors
Topological, compositional and information-theoretic network descriptors used in this study.

Total number of descriptors: 52, of which 27 are derivatives of others (resulting in 25 non-redundant descriptors)

1. Degree-related descriptors

These descriptors (except the last one) are calculated on the network biggest component. Total number of descriptors: 23

Number of Vertices [10] $n = |V|$, with V being all the nodes of the graph; nodes of degree 0 being excluded.

Number of Edges [10] $m = |E|$, with E being all the edges of the graph.

Average Vertex Degree [1–3, 6, 10] $\langle d_i \rangle$, with d_i being the degree of node i.

Total Adjacency [1–3, 6] $A = \sum_i d_i$

Connectedness / Density [1–3, 6] $\frac{A}{n(n-1)}$

Average Weighted Vertex Degree $\langle w_i \rangle$, with w_i being the weight of node i, calculated as the sum of the weights of the edges ending on this node. In the case of NIPs, the edge weight is the number of common metabolites for the pair of interacting pathways.
*(→ derivative of **Average Vertex Degree**)*

Total Weighted Adjacency $W = \sum_i w_i$
*(→ derivative of **Total Adjacency**)*

Weighted Connectedness $\frac{W}{n(n-1)}$
*(→ derivative of **Connectedness**)*

Average Clustering Coefficient $\langle cc_i \rangle$, with cc_i being the clustering coefficient of node i, calculated as

$$cc_i = \frac{2 \times |\{e_{jk}\}|}{d_i(d_i - 1)} : v_j, v_k \in N_i, e_{ij} \in E$$

(e_{jk} being an edge between v_j and v_k, and N_i being the nodes adjacent to v_i).

Average Weighted Clustering Coefficient $\langle cc_{i,w} \rangle$, with $cc_{i,w}$ being the weighted clustering coefficient of node i, calculated as

$$cc_{i,w} = \frac{2 \times \sum w_{jk}}{w_i(w_i - 1)} : v_j, v_k \in N_i$$

(w_{ij} being the weight of the edge $e_{ij} \in E$).
*(→ derivative of **Average Clustering Coefficient**)*

Total/Average/Normalized Information on Vertex Degree Distribution [2, 4, 5] Information content (several flavors[1]) of the vector defined as follows: for each possible node degree—from the lowest to the highest one found in all studied species network—the number of nodes having this degree in the current network (0 if none).
*(→ derivatives of **Information on Vertex Degree Distribution**)*

Total/Average/Normalized Information on Vertex Degree Magnitude Distribution [2, 4, 5] Information content (several flavors) of the vector defined as the list, in ascending manner, of all node degrees in the network.
*(→ derivatives of **Information on Vertex Degree Magnitude Distribution**)*

Total/Average/Normalized Information on Weighted Vertex Degree Distribution Information content (several flavors) of the vector defined as follows: for each possible node weight—from the lowest to the highest one found in all studied species network—the number of nodes having this weight in the current network (0 in none).
*(→ derivatives of **Information on Vertex Degree Distribution**)*

Total/Average/Normalized Information on Weighted Vertex Degree Magnitude Distribution Information content (several flavors) of the vector defined as the list, in ascending manner, of all node weights in the network.
*(→ derivatives of **Information on Vertex Degree Magnitude Distribution**)*

Number of Connected Components

2. Centrality-related descriptors

These descriptors are calculated on the biggest component only. Total number of descriptors: 7

Average Degree Centrality [9] $\langle dc_i \rangle$, with dc_i the fraction of vertices in the graph connected to node i,

$$dc_i = \frac{d_i}{|V| - 1}$$

Average Weighted Degree Centrality $\langle dc_{i,w} \rangle$, with $dc_{i,w}$ the fraction of weights of vertices connected to node i,

$$dc_{i,w} = \frac{w_i}{|V| - 1}$$

*(→ derivative of **Average Degree Centrality**)*

[1] A histogram with bins of size 1 is first derived for a given vector; S_i is the value of each bin i, and N_i its population. Three flavors of the information content of this vector (total, average and normalized) are then calculated as

$$IC_{total} = \text{NS} \log_2 \text{NS} - \sum_i N_i S_i \log_2 S_i$$

$$IC_{average} = \frac{IC_{total}}{\text{NS}} \quad ; \quad IC_{normalized} = \frac{IC_{total}}{\text{NS} \log_2 \text{NS}}$$

with $\text{NS} = \sum_i N_i S_i$

Average Vertex Betweenness Centrality [8, 9] $\langle vbc_i \rangle$, with vbc_i the fraction of number of shortests paths that go through node i,

$$vbc_i = \sum_{j \neq k \neq i \in V} \frac{|\sigma_{jk}(v_i)|}{|\sigma_{jk}|}$$

(σ_{jk} being the shortest paths from v_j to v_k and $\sigma_{jk}(v_i)$ the shortest paths from v_j to v_k going through v_i)

Average Weighted Vertex Betweenness Centrality $\langle vbc_{i,w} \rangle$, with $vbc_{i,w}$ the fraction of weights of shortests paths that go through node i,

$$vbc_{i,w} = \sum_{j \neq k \neq i \in V} \frac{\sum \omega_p(\sigma_{jk}(v_i))}{\sum \omega_p(\sigma_{jk})}$$

(the weight $\omega_p(\sigma_{jk})$ of a shortest path between v_j and v_k being the product of path weight over intermediate edges)
*(→ derivative of **Average Vertex Betweenness Centrality**)*

Average Edge Betweenness Centrality [9] $\langle ebc_i \rangle$, with ebc_i the fraction of shortest paths that go through the edge e_i,

$$ebc_i = \sum_{j \neq k \neq i \in V} \frac{|\sigma_{jk}(e_i)|}{|\sigma_{jk}|}$$

Average Weighted Edge Betweenness Centrality $\langle ebc_{i,w} \rangle$, with $ebc_{i,w}$ the fraction of shortest paths that go through the edge e_i,

$$ebc_{i,w} = \sum_{j \neq k \neq i \in V} \frac{\sum \omega_s(\sigma_{jk}(e_i))}{\sum \omega_s(\sigma_{jk})}$$

(the weight $\omega_s(\sigma_{jk})$ of a shortest path between v_j and v_k being the sum of path weight over intermediate edges)
*(→ derivative of **Average Edge Betweenness Centrality**)*

Average Closeness Centrality [9] $\langle clc_i \rangle$, with clc_i the inverse of the average distance from node i to all other nodes

3. Distance-related descriptors

These descriptors are calculated on the biggest component only. Total number of descriptors: 15
Total Graph Distance [6, 10] D, two times the sum of the shortest path length between each (ordered) pair of (connected) nodes.

Average Vertex Distance [1–3, 6] D/n

Average Graph Distance [1–3, 6] $\frac{D}{n(n-1)}$

Average Link Distance $\frac{D}{nm(n-1)}$

Total/Average/Normalized Information on Distance Degree Magnitude Distribution [2, 4–6] Information content (several flavors) of the vector defined as the list, in ascending order, of all distance degree (sum of distance to all other nodes) in the network.
*(→ derivatives of **Information on Distance Degree Magnitude Distribution**)*

Total/Average/Normalized Information on Distance Distribution [2, 4–7] Information content (several flavors) of the vector defined as follows: for each possible shortest path length between nodes—from the lowest to the highest one found in all studied species network—the number of shortest paths of this length in the current network (0 if none).
*(→ derivatives of **Information on Distance Distribution**)*

Total/Average/Normalized Information on Distance Magnitude Distribution [2, 4–7] Information content (several flavors) of the vector defined as the list, in ascending manner, of all shortest path lengths in the network
*(→ derivatives of **Information on Distance Magnitude Distribution**)*

Radius [2, 10] Minimum of all pairs shortest path length

Diameter [2, 10] Maximum of all pairs shortest path length

4. Cliques-related descriptors

For all the following descriptors, all cliques of size 2 are excluded. Total number of descriptors: 7
Total/Average/Normalized Information on Clique Distribution [4, 5] Information content (several flavors) of the vector defined as follows: for each possible clique size—from the lowest to the highest one found in all studied species network—the number of cliques of this size in the current network (0 if none).
*(→ derivatives of **Information on Clique Distribution**)*

Total/Average/Normalized Information on Clique Size Distribution [4, 5] Information content (several flavors) of the vector defined as the list, in ascending manner, of all clique sizes in the network
*(→ derivatives of **Information on Clique Size Distribution**)*

Number of Cliques [9]

5. *

References

[1] D. Bonchev. *Handbook of Proteomics Methods*, chapter Complexity of Protein-Protein Interaction Networks, Complexes and Pathways, pages 451–462. Humana, New York, 2003.

[2] D. Bonchev. Complexity analysis of yeast proteome network. *Chem Biodivers*, 1(2):312–326, 2004. ISSN 1612-1880 (Electronic). doi: 10.1002/cbdv.200490028.

[3] D. Bonchev. On the complexity of directed biological networks. *SAR QSAR Environ Res*, 14(3):199–214, 2003. ISSN 1062-936X (Print).

[4] D. Bonchev. *Information-Theoretic Indices for Characterization of Chemical Structures*. Research Studies Press, Chichester, 1983.

[5] D. Bonchev. *Mathematical Chemistry Series, Volume 7*, volume 7, chapter Shannon's Information and Complexity, pages 155–187. Taylor and Francis, 2003.

[6] D. Bonchev and G. Buck. *Complexity in Chemistry, Biology, and Ecology*, chapter Quantitative Measures of Network Complexity, pages 191–235. Springer, New York, 2005.

[7] D. Bonchev and N. Trinajstic. Information theory, distance matrix and molecular branching. *J Chem Phys*, 67:4517–4533, 1977.

[8] U. Brandes. A faster algorithm for betweenness centrality. Technical report, Department of Computer and Information Science, University of Konstanz, 2001. URL `citeseer.ist.psu.edu/brandes01faster.html`.

[9] *User Manual, NetMiner version 2.5.0*. Cyram, 2004.

[10] F. Harary. *Graph Theory*. Addison-Wesley, 1969.

[11] P. Jaccard. Nouvelles recherches sur la distribution florale. *Bull Soc Vaud Sci Nat*, (44):223–270, 1908.
