# Supplementary material for: Evolution of metabolic network organization
Source: BMC Syst Biol. 2010 May 11;4:59. doi: 10.1186/1752-0509-4-59 (PMC2876064; doi:10.1186/1752-0509-4-59)
Supplement: Additional file 6 — Classification models. List of the classification models with the best performance in discriminating taxa groups based on NIP descriptor values. [file 1752-0509-4-59-S6.PDF]

Additional file: Classification models
List of the classification models with the best performance in discriminating taxa groups based on NIP descriptor values.

Model	Description
Functions.LibSVM	Support vector machines classifier. Constructs a hyperplane—or set of hyperplanes—in a high or infinite dimensional space, which can be used for classification or regression [EL-Manzalawy, 2005, Chang and Lin, 2001]
Functions.Logistic	Builds a multinomial logistic regression model with a ridge estimator [le Cessie and van Houwelingen, 1992]
Functions.MultilayerPerceptron	Backpropagation neural network
Lazy.IB1	Nearest-neighbor classifier. Uses normalized Euclidean distance to find the training instance closest to the given test instance, and predicts the same class as this training instance. If multiple instances have the same (smallest) distance to the test instance, the first one found is used [Aha and Kibler, 1991]
Rules.JRip	Propositional rule learner, dubbed RIPPER—Repeated Incremental Pruning to Produce Error Reduction [Cohen, 1995]
Rules.OneR	One-rule classifier. Uses the minimum-error attribute for prediction, discretizing numeric attributes [Holte, 1993]
Trees.RandomForest	Constructs a forest of random trees [Breiman, 2001]

References

D. Aha and D. Kibler. Instance-based learning algorithms. *Machine Learning*, 6: 37–66, 1991.

L. Breiman. Random forests. *Machine Learning*, 45(1):5–32, 2001.

C.-C. Chang and C.-J. Lin. Libsvm - a library for support vector machines, 2001. URL `http://www.csie.ntu.edu.tw/ cjlin/libsvm/`. The Weka classifier works with version 2.82 of LIBSVM.

W. W. Cohen. Fast effective rule induction. In *Twelfth International Conference on Machine Learning*, pages 115–123. Morgan Kaufmann, 1995.

Y. EL-Manzalawy. Wlsvm, 2005. URL `http://www.cs.iastate.edu/ yasser/wlsvm/`.

R. Holte. Very simple classification rules perform well on most commonly used datasets. *Machine Learning*, 11:63–91, 1993.

S. le Cessie and J. van Houwelingen. Ridge estimators in logistic regression. *Applied Statistics*, 41(1):191–201, 1992.
